# The Importance of Patency in Patients with Critical Limb Ischemia Undergoing Endovascular Revascularization for Infrapopliteal Arterial Disease

**DOI:** 10.3389/fcvm.2014.00017

**Published:** 2015-01-07

**Authors:** Frederic Baumann, Christoph Ozdoba, Ernst Gröchenig, Nicolas Diehm

**Affiliations:** ^1^Miami Cardiac and Vascular Institute, Baptist Hospital, Miami, FL, USA; ^2^Clinical and Interventional Neuroradiology, University Hospital Bern, Bern, Switzerland; ^3^Clinical and Interventional Angiology, Kantonsspital Aarau, Aarau, Switzerland; ^4^University of Applied Sciences Furtwangen, Villingen-Schwenningen, Germany

**Keywords:** critical limb ischemia, below the knee, endovascular revascularization, patency

## Abstract

Critical limb ischemia (CLI) represents the most severe form of peripheral arterial disease (PAD) and frequently occurs in medically frail patients. CLI patients frequently exhibit multi-segmental PAD commonly including the tibial arterial segment. Endovascular therapy has been established as first-line revascularization strategy for most CLI patients. Restenosis was reported to occur in up to more than two-thirds of CLI patients undergoing angioplasty of complex tibial arterial obstructions. Nevertheless, favorable clinical outcomes were observed for infrapopliteal angioplasty when compared with bypass surgery, despite higher patency rates for the latter. Based on these observations, infrapopliteal patency was considered to be only of secondary importance upon clinical outcomes in CLI patients. In contrast to these earlier observations, however, recent findings from two randomized clinical trials indicate that infrapopliteal patency does impact on clinical outcomes in CLI patients. The purpose of the present manuscript is to provide a critical reappraisal of the present literature on the clinical importance of tibial arterial patency in CLI patients undergoing endovascular revascularization and to discuss utility and limitations of currently available anti-restenosis technologies.

## Introduction

Critical limb ischemia (CLI) represents a medically frail subgroup of patients presenting with the most severe form of peripheral arterial disease (PAD) (1–3). In these patients, revascularization is among the cornerstones of treatment and aims at the prevention of amputation and improvement of quality of life (1, 4, 5). CLI patients frequently present with multilevel PAD including complex obstructions of tibial arteries and, thus, frequently require challenging revascularization procedures. Endovascular therapy has been established as a first-line revascularization strategy for most CLI patients (5, 6) since it was shown to provide comparable clinical outcomes when compared with bypass surgery, despite higher patency rates for the latter (5, 7). Based on these observations, the tide over concept was established assuming that tibial arterial patency was mandatory only during the process of wound healing but not thereafter for the maintenance of skin integrity and resolution of CLI symptoms (7–9). More recent studies, however, have stressed the importance of tibial patency upon clinical outcomes in CLI patients, thereby challenging the tide over concept (10–13).

The purpose of this review article is to provide a critical reappraisal of the importance of tibial arterial patency on clinical outcomes in CLI patients. Moreover, we seek to analyze recently performed trials on endovascular revascularization for tibial PAD and to stress their applicability to everyday clinical practice.

## Methods

A comprehensive literature research was performed based on Pubmed database. All studies included in the meta-analysis by Romiti et al. (7) were acquired using Pubmed and analyzed thereafter (researched April 2013). In addition, we reviewed literature for completed and ongoing randomized trials on drug-eluting stents (DESs) and drug-eluting balloon (DEB) versus bare metal stent (BMS) and/or plain-old balloon angioplasty (POBA) for tibial arterial revascularization. The latter literature research was based on Pubmed (www.pubmed.org, last accessed on March 13, 2014) and clinicaltrials.gov (last accessed on April 28, 2014) entries.

## Incidence of Tibial Arterial Restenosis after Angioplasty

Restenosis remains the major drawback in CLI patients undergoing endovascular therapy of tibial arterial obstructions. Various studies reported tibial restenosis to occur in up to more than two-thirds of patients undergoing angioplasty of complex tibial arterial obstructions (10–12, 14, 15). Within a prospective study, Schmidt and colleagues evaluated the incidence of tibial arterial restenosis in CLI patients undergoing POBA. Restenosis was defined as a lumen compromise ≥ 50% on serial angiography after 3 months (15). A total of 58 CLI patients (77 limbs) with a mean tibial lesion length of 184 mm were analyzed. In that cohort, binary restenosis was observed in 68.8% of limbs. Similar results were observed by Liistro and coworkers within the DEBATE-BTK trial (10) comparing POBA versus DEB. In that study, binary restenosis was assessed by angiography and defined as a reduction of luminal diameter > 50% or by duplex sonography defined as a peak systolic velocity index ≥ 2.5. After 1 year, tibial restenosis was observed in 74% in the POBA (74 limbs, mean lesion length: 131 mm) and 27% in the DEB group (74 limbs, mean lesion length: 129 mm [*P* < 0.001]). In addition, Iida and coworkers analyzed the incidence of tibial restenosis and its impact on clinical outcomes in CLI patients after POBA in a total of 63 patients (12). Restenosis was evaluated angiographically and defined as a reduction of luminal diameter ≥ 50%. After 3 months, tibial restenosis was observed in 74/102 (73%) of treated lesions. Of note, no detailed information on tibial lesion length was provided in that study, although tibial lesion length was shown to be indicative for the risk of tibial restenosis (16).

## Impact of Tibial Arterial Patency on Clinical Outcomes in CLI

Despite the high rates of tibial restenosis subsequent to POBA, endovascular revascularization is considered the first-line treatment strategy for most CLI patients (5, 6). Until now, the BASIL trial was the first and only trial randomly comparing endovascular therapy with open surgery in CLI patients undergoing infrainguinal revascularization (5). In that trial, a total of 452 patients (224 angioplasty, 228 surgery) were analyzed for amputation-free survival. After 1 year, an amputation-free survival was obtained in 68 versus 71% and after 3 years 57 versus 53% comparing bypass surgery with angioplasty (*P* > 0.05). This observation was underlined by a frequently cited meta-analysis by Romiti et al. (7). In that meta-analysis, the impact of tibial patency on clinical outcomes comparing endovascular versus surgical revascularization strategies in CLI was scrutinized. For that reason, a total of 30 studies including 2646 patients were analyzed (Table 1). Primary patency was 58.1, 51.3, and 48.6% after tibial angioplasty and 81.5, 76.8, and 72.3% after tibial bypass surgery at 12, 24, and 36 months (*P* < 0.05). Of interest, despite these significant differences in patency rates, no significant differences in clinical outcomes were observed: limb salvage rates were 86.0, 83.8, and 82.4% in patients treated by angioplasty and 88.5, 85.2, and 82.3% after tibial bypass surgery at same intervals. Based on these findings, the tide over concept was established assuming that increased perfusion was mandatory for ulcer healing in CLI but not thereafter for maintaining skin integrity. Therefore, tibial arterial patency was considered to be of minor importance during mid- and long-term follow-up of CLI patients.

**Table 1:** **Summary of all studies analyzed within the meta-analysis of Romiti and coworkers**.

Ref.	Patients (*n*)	Limbs (n)	s/e	Patency evaluation (specifications)	End points	Reported fu (months)	Mean fu (months)
Haider et al. (17)	32	32	e	DUS	PP: 60%	24	n.i.
Kudo et al. (18)	52	52	e	DUS/ABI	PP: 23.5%, SP: 46.1%, LS: 77.3%	36	14.7
Boyer et al. (19)	49	49	e	DUS	PP: 81%, SP: 88%, LS: 87%	36	21
Parsons et al. (20)	66	66	e	ABI/pulse volume recordings	PP: < 15%	12	n.i.
Spinosa et al. (21)	37	37	e	ABI/pulse volume recordings	LS: 66%	12	7.8
					No info on patency	
Wölfle et al. (22)	s:125	130, IC: 3, CLI: 127	s/e	CI/ABI (DUS after 1991)	PPs: 46%, SPs: 49%, LSs: 63%	84 (s)	n.i.
	e:84	89, IC: 5, CLI: 84			LSe: 63%, e: no patency information	72 (e)	n.i.
Marzelle et al. (23)	23	23	e	Clinical	PP: 34%, LS: 71%	12	8.6
Vraux et al. (24)	36	40	e	DUS	PP: 56%, SP: 72%, LS: 81%	12	10
Treiman et al. (25)	25, IC: 5, CLI: 20	25	e	CI: ABI, DUS/angiography, (if ABI-impair > 0.1 or clinical deterioration)	CI: 59% (32%, 20%)	12 (24, 36)	44
Brosi et al. (26)	29	38, IC: 13, CLI: 25	e	ABI/clinical	LS: 73%	12	5.9
Aulivola et al. (27)	79	90	e	n.i.	LS: 84.4% (52.5%) non-ESRD, LS: 80.2% (52.5%) ESRD	(12, 36)	14.3
Sigala et al. (28)	50	50	e	Clinical	LS: 68.9%	24	15
Brillu et al. (29)	37	37	e	Clinical	LS: 87%	24	28
Brown et al. (30)	40	55	e	CI	CI: 44%	25.8	25.8
Bull et al. (31)	168, IC: 40, CLI: 128	168	e	CI	CI: 83% (single stenosis), CI: 76% (multilevel lesions), CI: 44% (lytic therapy), CI: 36% (segmental occlusion)	36	26.1
Danielsson et al. (32)	140	155, IC: 16, CLI: 139	e	CI (improvement of subjective relief)	CI: 66% (non-DM) CI: 32% (DM), LS: 66% (non-DM), LS: 90% (DM)	12	n.i.
Favre et al. (33)	24, IC: 4, CLI: 20	25	e	DUS	PP: 46%, SP: 64%	24	15
Löfberg et al. (34)	82	86	e	CP (according to SVS/ISCVS standards)	CP: 36%, LS: 72%	36, 36	n.i.
Ingle et al. (35)	67, IC: 6, CLI: 61	70	e	CP (freedom from CLI)	CP: 84%, LS: 94%,	36	n.i.
Vraux et al. (24)	46	50	e	Intention to treat CP	PP: 46%, SP: 55%, CP: 63%, LS: 87%	12	15
Nydahl et al. (36)	27, IC: 4, CLI: 24	28	e	CP (symptomatic patency)	CP: 56%, LS: 85%, survival: 81%	12	n.i.
Tisi et al. (37)	57	57	e	DUS: *n* = 26, Angiography: *n* = 3 (angiography if ABI-impair > 0.1 or clinical deterioration)	PP: 27%, SP: 33%, LS: 88%	12	n.i.
Söder et al. (38)	60	72	e	Angiography	PP: 48%, SP: 56%, LS 80%	18	10
Barton et al. (39)	43	n.i.	e	CI (asymptomatic)	CI: 60%	36	28
Lazaris et al. (40)	24	24	e	Intention to treat	PP: 50%, LS: 92%	12	n.i.
Sivananthan et al. (41)	38, IC: 18, CLI: 20	41	e	CI: (improvement ≥ 1 Fontaine category)	CI: 58%	at last fu	21
Faglia et al. (8)	537, s: 117, e: 420	537	s/e	CP (no recurrence of pain/ulcer)	CP, PTA: 78%, Bypass: 77%	60	40
Bosiers et al. (42)	443	443	e	DUS	PP: 74.2%, LS: 96.6%	12, 12	n.i.
Schwarten (43)	96	112	e	n.i.	LS: 83%	24	n.i.
Ascher et al. (44)	30	32, IC: 12, CLI: 20	e	DUS	LS: 100%, PP: 85%	3	5.2

For several reasons, however, the validity of the methodological design and conclusion of that meta-analysis must be considered as limited. First, the sample size in the majority of studies included was small and ranged from 23 to 537 patients. Of note, 16/30 (53.3%) studies had included less than 50 patients. In addition, 9/30 (30%) studies included both CLI and patients with intermittent claudication. Thus, a substantial fraction of the patients (548/2646, 20.7%) did not suffer from limb-threatening ischemia. Accordingly, statistical power of both individual studies and the meta-analysis was limited. Second, a direct comparison of functional clinical outcomes is limited due to substantial variability of clinical end point definitions or the lack of clinical outcome reports at all (20, 33). Clinical end point definitions included subjective relief, freedom from CLI, improvement of clinical classification, and limb salvage (8, 32, 40, 41). Moreover, clinical outcomes were not reported throughout all studies but only in 26/30 (86.7%) studies. Third, systematic patency evaluation was performed in only 9/30 (30%) of studies included within the meta-analysis (17–19, 24, 33, 37, 38, 44, 45). Remarkably, patency was assessed by duplex sonography in 8/30 (26.7%) studies and by angiography in only 1 (3.3%) study in a total of 60/2646 (2.3%) patients. Thus, the vast majority of patency evaluation was performed by duplex sonography, although its validity in tibial arteries is highly controversial (42). In addition, information on tibial patency was derived from the clinical need for repeated intervention in 4/30 (13.3%) studies. Thus, arterial patency rates may have been overestimated utilizing this surrogate definition. Fourth, Romiti et al. published 3-year outcomes, although only 16/30 (53.3%) studies reported follow-up results beyond 24 months.

In addition to these limitations, the importance of tibial patency upon clinical outcomes was recently endorsed by various studies (10–13). It was shown that CLI patients require frequently target lesion revascularization (TLR) to maintain favorable clinical results subsequent to tibial angioplasty. Within the aforementioned study by Iida, TLR was necessary in 48% of patients with documented restenosis of the tibial target lesion in 73% at 12-month follow-up. In addition, the authors observed a prolonged time of wound healing in patients with tibial restenosis when compared to patients without restenosis: 127 versus 66 days (*P* = 0.02). The high prevalence of tibial TLR was recently corroborated by Baumann et al. within a consecutive series of 128 CLI patients undergoing tibial angioplasty (13). That group aimed for a comparison analyzing the clinically driven need for TLR versus target extremity revascularization (TER) after tibial angioplasty. After 1 year, TLR was performed in 41.6% and TER in 17.2% of patients. While adding proof to the high prevalence of TLR after tibial angioplasty, that observation moreover indicated that tibial restenosis is of greater clinical impact than progression of atherosclerotic disease as reflected by TER rates.

Moreover, Rastan et al. were the first to underline the importance of tibial patency within a randomized setting (11). The Yukon BTK trial randomly assigned a total of 161 patients comparing DES (82 patients) to BMS (79 patients) for tibial angioplasty in CLI. Primary clinical end point in the Yukon BTK trial was an event-free survival defined as freedom from target limb amputation, target vessel revascularization, myocardial infarction, and death. After a follow-up period of 1016 days, an event-free survival was attained in 65.8% in the DES group versus in 44.6% in the BMS group (*P* = 0.02). In line with clinical observations, primary tibial patency at 1 year was 80.6 versus 55.6% (*P* = 0.004) when comparing DES with BMS (46).

Thus, in consideration of these substantial limitations behind the tide over concept and given observations from more recent clinical trials (11–13), the ultimate importance of tibial arterial patency subsequent to endovascular therapy remains to be determined.

## Currently Available Technologies Aimed at the Prevention of Restenosis

Given the excessive incidence of tibial arterial restenosis (10, 12, 14, 15) subsequent to POBA, various endovascular technologies have been assessed in the framework of clinical trials.

## Bare Metal Stents

Mechanical scaffolding as provided by a stent may be an ideal solution to address elastic recoil, an important contributor to restenosis in tibial arteries (47). The application of tibial BMS was assessed in various studies (48–50). However, no substantial benefit of BMS application when compared with POBA was observed within three randomized trials (48–50) (Table 2). Based on these observations, it may be assumed that neointimal proliferation induced by BMS outweighs the potential benefit of mechanical scaffolding in the prevention of restenosis induced by elastic recoil.

**Table 2:** **Overview of randomized series comparing BMS with POBA for tibial revascularization in CLI patients**.

Ref.	No. patients/lesions	Lesion length (mm)	Follow-up	Patency evaluation (number)	Patency (%)	Clinical end points (%)
**COMPLETED RANDOMIZED TRIALS ON BMS FOR BTK**						
Rand et al. (49)	51/95	24	6 months	Angiography: 18	BMS: 79.7	LS
	BMS^a^: 42			BMS: 9	PTA 45.6 (*P* = 0.02)	BMS: 92
	PTA: 53			PTA: 9		PTA: 95 (*P* = ns)
				CT-Angio: 19	
				BMS: 8		
				PTA: 11	
Randon et al. (50)	35/38	BMS: 22	12 months	Clinical patency	BMS: 66.0	LS
	BMS^b^: 16	PTA: 39			PTA: 79.5 (*P* = ni)	BMS 92.7
	PTA: 22					PTA: 90.0 (*P* = 0.76)
Brodmann et al. (48)	54/54	BMS: 28	12 months		BMS: 35.3	CI
	BMS^a^: 21	PTA: 79			PTA: 41.8 (*P* = ns)	BMS: 64.7
	PTA: 33					PTA: 81.5 (*P* = ns)
**PLANNED OR ONGOING RANDOMIZED TRIALS ON BMS FOR BTK ANGIOPLASTY**						
XXS^c^	180	<150	12	Angiography	–	TLR

*BMS, bare metal stent; BTK, below the knee; No., number; PTA, percutaneous angioplasty; ns, not significant; ni, no information; LS, limb salvage; CI, clinical improvement (improvement ≥ 1 category according to Rutherford classification); *P, P* value; TLR, target lesion revascularization*.

*^a^Balloon-expandable BMS*.

*^b^Including balloon-expandable and self-expandable BMS*.

*^c^Self-expandable BMS*.

## Drug-Eluting Stents

Given the above-outlined drawbacks of BMS in tibial interventions, great hope was attributed to DES technology. The principle of DES is to provide mechanical scaffolding but with a minimum of neointimal proliferation based on the antiproliferative coating. Four randomized trials compared DES versus POBA or BMS for tibial angioplasty (46, 51–53) (Table 3). Whilst the Yukon trial compared DES with BMS (46), the remaining randomized studies compared DES to POBA (51–53). Without exception, DES was superior when compared with BMS/POBA for tibial angioplasty in respect to patency and the need for repeated TLR. In addition, DES was shown to improve event-free survival rates when compared with BMS as shown within the aforementioned Yukon trial (11).

**Table 3:** **Overview of randomized trials comparing DES versus BMS or POBA for BTK angioplasty**.

Reference	Devices	Rutherford categories	Renal insufficiency	Inclusion criteria	Patients (*n*)	Follow-up (months)	Final LL	End point	Results
Yukon (46)	DES° versus BMS (° Yukon, Translumina, Hechingen, Germany)	2–5	n.i.	*de novo* lesions stenosis > 70%, LL < 45 mm	161	12	31 ± 9	Restenosis (>50%) (a) DUS (PSVR > 2.4)	**Primary patency**
									DES: 80.6%
									BMS: 55.6% (*P* = 0.004)
									**Secondary patency**
								(b) Angiography	DES: 91.9%
									BMS: 71.4% (*P*:0.005)
Destiny (52)	DES° versus BMS (° Xience V stent)	4, 5	n.i.	*de novo* stenosis > 50%, LL < 40 mm	140	12	n.i.	Restenosis > 50% by angiography	**Primary patency**
									DES: 85%
									BMS: 54% (*P* = 0.0001)
Falkowski (53)	DES° versus BMS (° Cipher Cordis Europa N.V.)	3–5	n.i.	*de novo* stenosis > 60%, LL 5–30 mm	50	6	17.8	PE: Restenosis > 50% by angiography	**Primary patency**
									DES: 16%
									BMS: 76% (*P* = 0.001)
								SE: TLR	**TLR**
									DES: 12%
									BMS: 56% (*P* < 0.05)
**RANDOMIZED TRIALS ON DES VERSUS POBA FOR BTK ANGIOPLASTY**									
Achilles (51)	DES° versus POBA (° Cipher Select, Cordis Cooperation, USA)	3–5	exclusion: creatinine > 2.5 mg/dl	*de novo* and restenotic native stenosis > 70%, LL < 120 mm	200 (99 versus 101)	12	27 ± 21	Restenosis by angiography	**Primary patency**
									DES: 77.6%
									POBA: 58.1%

*n, number; n.i., no information; LL, lesion length; DUS, duplex ultrasound; PSVR, peak systolic velocity ratio; DES, drug-eluting stent; BMS, bare metal stent; TLR, target lesion revascularization; PE, primary end point; SE, secondary end point; POBA, plain-old balloon angioplasty*.

Of note, however, tibial arterial lesion lengths of patients included in all randomized DES trials were ≤35 mm. Within a consecutive series, Baumann et al. analyzed tibial lesion morphology in 105 CLI patients undergoing tibial angioplasty (16). Thereby, a mean lesion length of 87 mm was observed for stenotic and 124 mm for occlusive tibial PAD. According to these morphological findings, only 11% of that study population would have qualified for participation within the above-mentioned randomized DES trials. Thus, currently available coronary DES is applicable to only a minority of CLI patients treated in everyday clinical practice.

## Drug-Eluting Balloons

DEB technology was introduced with the intention of reducing neointimal proliferation. First, by limiting the mechanical irritation to the duration of balloon inflation, and second, by the application of an antiproliferative substance during the endothelial injury phase induced by angioplasty. According to first observations, the application of DEB may reduce restenosis and the need for repeated revascularization when compared with POBA for tibial revascularization (10, 54).

Within a non-randomized study setting, Schmidt et al. evaluated the application of DEB for tibial revascularization in 104 patients (CLI: 82.6%, severe claudication: 17.4%, limbs: n = 109) (54). Binary restenosis was evaluated using angiography and defined as a > 50% reduction of lumen diameter. After 3 months, binary restenosis after DEB was observed in 27.4%. Within a similar cohort of historic control patients at the same center undergoing tibial POBA, restenosis was reported in 68.8% after 3 months (54). According to this, DEB was shown to reduce tibial restenosis by around 60% when compared with POBA and, thus, great hope was placed on DEB technology aimed at improving tibial patency. The superiority of DEB over POBA in tibial arteries was furthermore shown by Liistro et al. who were the first to report results from a randomized trial (10). Within the DEBATE-BTK trial, Liistro and coworkers analyzed 132 patients for tibial angioplasty randomly assigned for 67 DEB and 65 POBA. Mean lesion length was 129 mm in patients treated with DEB and 131 mm treated with POBA (*P* = 0.9). Primary end point in that trial was binary restenosis defined > 50% after 12 months by angiography (>90% of patients) and/or duplex sonography for the remaining. Secondary clinical end points were the incidence of TLR and amputation. Binary restenosis was 27% in the DEB and 74% in the POBA group (*P* < 0.001). In addition, the need for secondary TLR was lower in patients treated with DEB compared to those treated with POBA (18 versus 43%, *P* = 0.002).

Of note, for technical reasons, operators within most of the randomized and observational trials including the DEBATE-BTK trial were not blinded to treatment allocation. This may have influenced the decision of performing TLR and may serve as an explanation for the wide range of TLR rates. During 1-year follow-up, amputation was necessary in one patient in the POBA group and none in the DEB group (*P* = 0.9). In the meantime, results from further randomized trials are awaited (Table 4). Of these, the Euro Canal trial was terminated early due to strategic reorientation of the company. A second randomized trial performed was the InPact Deep trial, which was finished but upon completion the company withdrew the DEB of investigation from the market. This was based on the 12-month results with lacking efficacy of DEB and moreover higher major amputation rates for DEB (8.8%) when compared to POBA (3.6%, *P* = 0.08).

**Table 4:** **Overview of ongoing/not completed randomized trials on DEB versus BMS or POBA for BTK angioplasty**.

Study name	Devices	Rutherford categories	Predefined LL (mm)	Patients (*n*)	Follow-up (months)	End points
**PLANNED/ONGOING RANDOMIZED TRIALS ON DEB FOR BTK ANGIOPLASTY**						
IDEAS-I	DEB versus BMS	3–6	70–220	50	6	Restenosis (angiography)
Piccolo	DEB versus POBA	3–5	15–150	114	6	Late lumen loss (angiography)
InPact Deep^a^	DEB versus POBA	4–6	<100	450	12	Clinically driven TLR, restenosis (angiography)
Euro Canal^b^	DEB versus POBA	4–6	10–270	120	6	Late lumen loss (angiography)

*DEB, drug-eluting balloon; BMS, bare metal stent; POBA, plain-old balloon angioplasty; BTK, below the knee; LL, lesion length; n, number; TLR, target lesion revascularization; DUS, duplex ultrasound*.

*^a^Study terminated early based on safety concerns*.

*^b^Study terminated early based on strategic company decision, no safety concerns*.

## Conclusion

In contrast to earlier observations, patency appears to affect clinical outcomes in CLI patients, and thus, remains the major drawback of tibial arterial angioplasty. DES and DEB were shown to improve tibial patency but both with specific limitations. Accordingly, currently applied and evaluated DES for tibial revascularization do not address infrapopliteal lesion morphology. While DEB technology complies well with tibial lesion morphology in CLI patients, it may not address acute elastic recoil, an important contributor to tibial restenosis. Further studies assessing anti-restenosis concepts specifically dedicated to the unique requirements of complex tibial arterial obstructions are warranted.

## Conflict of Interest Statement

Nicolas Diehm was the Principal Investigator for Switzerland of the Euro Canal trial and received consulting honoraria from Medrad. This trial, however, was terminated early due to strategical reorientation of the company. Thereafter, Nicolas Diehm has no more financial disclosures or conflicts of interest to report. The remaining authors have neither financial disclosures nor a conflict of interest to state.
